# The genetic risk of acute seizures in African children with falciparum malaria

**DOI:** 10.1111/epi.12173

**Published:** 2013-04-24

**Authors:** Symon M Kariuki, Kirk Rockett, Taane G Clark, Hugh Reyburn, Tsiri Agbenyega, Terrie E Taylor, Gretchen L Birbeck, Thomas N Williams, Charles R J C Newton

**Affiliations:** * Kenya Medical Research Institute, Centre for Geographic Medicine Research CoastKilifi, Kenya; † Department of Psychiatry, University of OxfordOxford, United Kingdom; ‡ Wellcome Trust Centre for Human Genetics, University of OxfordOxford, United Kingdom; § Faculty of Infectious and Tropical Diseases, London School of Hygiene and Tropical MedicineLondon, United Kingdom; ¶ Department of Physiology, School of Medical Sciences, Kwame Nkurumah University of Science and TechnologyKumasi, Ghana; # Department of Internal Medicine, College of Osteopathic Medicine, Michigan State UniversityEast Lansing, Michigan, U.S.A; ** International Neurologic and Psychiatric Epidemiology Program, Michigan State UniversityEast Lansing, Michigan, U.S.A; †† Nuffield Department of Clinical Medicine, University of OxfordOxford, United Kingdom; ‡‡ Neurosciences Unit, Institute of Child Health, University College LondonLondon, United Kingdom

**Keywords:** Africa, Children, Seizure phenotypes, Malaria-associated seizures, Genetic risk

## Abstract

**Purpose:**

It is unclear why some children with falciparum malaria develop acute seizures and what determines the phenotype of seizures. We sought to determine if polymorphisms of malaria candidate genes are associated with acute seizures.

**Methods:**

Logistic regression was used to investigate genetic associations with malaria-associated seizures (MAS) and complex MAS (repetitive, prolonged, or focal seizures) in four MalariaGEN African sites, namely: Blantyre, Malawi; Kilifi, Kenya; Kumasi, Ghana; and Muheza, Tanzania. The analysis was repeated for five inheritance models (dominant, heterozygous, recessive, additive, and general) and adjusted for potential confounders and multiple testing.

**Key Findings:**

Complex phenotypes of seizures constituted 71% of all admissions with MAS across the sites. MAS were strongly associated with cluster of differentiation-ligand-rs3092945 in females in Kilifi (p = 0.00068) and interleukin (IL)-17 receptor E-rs708567 in the pooled analysis across the sites (p = 0.00709). Complex MAS were strongly associated with epidermal growth factor module-containing mucin-like hormone receptor (EMR)1-rs373533 in Kumasi (p = 0.00033), but none in the pooled analysis. Focal MAS were strongly associated with IL-20 receptor A-rs1555498 in Muheza (p = 0.00016), but none in the pooled analysis. Prolonged MAS were strongly associated with complement receptor 1-rs17047660 in Kilifi (p = 0.00121) and glucose-6-phosphate dehydrogenase-rs1050828 in females in the pooled analysis (p = 0.00155). Repetitive MAS were strongly associated with EMR1-rs373533 in Kumasi (p = 0.00003) and cystic fibrosis transmembrane conductance receptor-rs17140229 in the pooled analysis (p = 0.00543). MAS with coma/cerebral malaria were strongly associated with EMR1-rs373533 in Kumasi (p = 0.00019) and IL10-rs3024500 in the pooled analysis across the sites (p = 0.00064).

**Significance:**

We have identified a number of genetic associations that may explain the risk of seizures in >2,000 cases admitted to hospitals with MAS across four sites in Africa. These associations differed according to phenotype of seizures and site.

Acute seizures are common in children admitted with falciparum malaria to hospitals in sub-Saharan Africa (Taylor et al., 2006; Idro et al., 2008). Some malarial seizures are focal, repetitive, or prolonged (complex), which are associated with the development of subsequent neurocognitive impairments or epilepsy (Carter et al., 2005; Birbeck et al., 2010). It is not clear if these seizures are febrile seizures or acute symptomatic seizures caused by the sequestration of the parasites within the brain (Idro et al., 2005). Furthermore, it is difficult to attribute seizures to malaria in a malaria-endemic area, since a proportion of children will have sequestered parasites and be asymptomatic, and malaria is the most common cause of fever in children aged 6 months to 6 years (Idro et al., 2005). We have determined that most seizures in children admitted to hospital with malaria parasitemia are attributable to malaria using logistic regression techniques (Kariuki et al., 2011), and that children admitted with seizures are more likely to have relatives with seizure disorders than controls (Versteeg et al., 2003).

There is a strong genetic predisposition for febrile seizures, investigated through twin studies, family history, and a number of genetic polymorphisms (DNA sequence variation occurring when a single nucleotide—A, T, C, or G—n the genome differs between members of a biologic species; Rich et al., 1987; Kugler & Johnson, 1998; Kjeldsen et al., 2002). The risk of febrile seizures has been associated with interleukin-1β (IL-1B; Virta et al., 2002; Kira et al., 2005) and interleukin-1 receptor antagonist (IL-1RA; Tsai, 1987). Furthermore, a number of polymorphisms associated with falciparum malaria infections have been identified, which may be protective for example, sickle cell trait (Williams et al., 2005), glucose-6-phosphate dehydrogenase (G6PD; Clark et al., 2009), interadhesion molecule (ICAM-1; Kun et al., 1999), and cluster of differentiation ligand (CD-40LG; Sabeti et al., 2002), or susceptible, for example, complement receptor (CR-1; Nagayasu et al., 2001) and IL-10, -12, and -18 (Chaisavaneeyakorn et al., 2003; Wilson et al., 2005). However, the role of these genes in the genesis of seizures has not been examined.

We examined a number of different polymorphisms associated with malaria-associated seizures (MAS), and the phenotypes of MAS using 4,472 children admitted to hospitals with falciparum malaria in four African countries (Ghana, Kenya, Malawi, and Tanzania) as part of a case–control study for MalariaGEN (Malaria Genetic Epidemiological Network) consortial project (MalariaGEN, 2008). The malaria candidate polymorphisms in the MalariaGEN consortial project (Table S1) were chosen for this analysis because they reflect a pathogenic mechanism for severe malaria that could be involved in the genesis of acute seizures in falciparum malaria. Malaria pathogenesis is considered in the context of malaria with impaired consciousness and/or respiratory distress (Marsh et al., 1996), although the former (of which cerebral malaria is the most important severe neurologic complication) is important as seizures occur in >80% of the cases (Idro et al., 2005).

## Methods

### Study sites, participants, and samples

The study participants for this analysis were recruited from four of the sites contributing date to the MalariaGEN study as described in detail elsewhere (MalariaGEN, 2008). The four sites were Queen Elizabeth Central Hospital, Blantyre, Malawi; Kilifi District Hospital on the coast of Kenya; Teule Hospital in Moshi, Tanzania; and Komfo Anokye Teaching Hospital in Kumasi, Ghana.

The entomologic inoculation rate (EIR) for *P. falciparum* in the city of Blantyre is estimated to be around one infective bite/person/year, but a high proportion of families make regular visits to nearby rural areas where the EIR is estimated to be >100 (Table S2). In Kilifi, the overall annual EIR has been estimated at 1–100, and recent years have seen a significant decline in the rate of transmission from mesoendemic in the 1990s to hypoendemic transmission today. In Kumasi, Annual Biting Rates and Annual Entomological Inoculation Rates have been reported to be 11,643 and 866, respectively. Muheza has an intense transmission of *P. falciparum* (50–700 infected bites/person/year), with two seasonal peaks and the community prevalence of *P. falciparum* in children aged 2–5 years in the study area recorded as high as 88.2% in 2002. Additional detailed description of the sites is provided in Table S2.

Detailed data were available for each study participant, including a range of demographic parameters. We defined cases as children admitted to hospital with severe malaria with a history of seizures during the acute illness, and controls as children admitted with severe malaria without a history of seizures during their life. Severe malaria was diagnosed as the presence of peripheral parasitemia in the blood of a child presenting with coma or impaired consciousness, respiratory distress, acidosis (base excess less than −8), hypoglycemia (whole blood glucose < 2.2 mm), and/or severe normocytic anemia (<5 g/dl), with exclusion of other coincidental causes of severe illnesses such as pneumonia (Marsh et al., 1995; World Health Organization, 2000). The blood was put on a slide stained with 10% *Giemsa*. The distribution of cases and controls, in that order, was 770 and 620 in Blantyre, 900 and 1,148 in Kilifi, 250 and 250 in Kumasi, and 175 and 359 in Muheza. Children in this study were treated appropriately according to the local national guidelines and protocols.

### Case definitions of phenotypes of MAS

Children with MAS (whose DNA samples were available for genotyping) admitted to hospital between 1996 and 2001 in Blantyre, 2002 and 2008 in Kilifi, 2006 and 2009 in Kumasi, and 2006 and 2007 in Muheza were selected. Details about various seizure semiologies and or malaria were extracted from the clinical notes and databases at each site, and the notes were further examined in order to categorize cases and controls.

We classified MAS into two major phenotypes namely: simple and complex, with complex further subdivided into focal MAS, repetitive MAS, and prolonged MAS (Table 1), based upon the International League Against Epilepsy definitions for acute/febrile seizures (ILAE, 1989, 1993). Focal MAS were defined as convulsions, twitches, or subtle seizures (cycling or suckling movements) starting in one part of the body or associated with lateralized asymmetric body weakness (Todd's paresis). Repetitive seizures were defined as two or more seizures occurring within 24 h of an illness or during the same illness. Seizures occurring in the ward were considered prolonged if they were lasting ≥15 min as documented by a clinician or a nurse, or phenytoin or phenobarbital was needed to stop uncontrolled seizures, or if seizures on admission did not stop after the first dose of diazepam (Sadarangani et al., 2008). Seizures were also considered prolonged if the histories from parents or caretakers, who rarely have watches, suggested that they lasted for ≥15 min, that is, seizures lasting the period of the journey to the hospital for those living more than a kilometer away, or seizures lasting more than the period of boiling a pot of maize, milking a dairy cow, or lasting more than the news broadcast on the radio. The “all focal,” “all repetitive” or “all prolonged” included any complex phenotype and were not mutually exclusive (Gwer et al., 2012). Simple seizures included those that were short (<15 min or if parental history as explained above suggested so), generalized and occurred once during the episode of malaria.

**Table 1:** Case definition characterizing the phenotypes of malaria-associated seizures

Category of seizures	Case definition characterizing the seizure phenotype
Repetitive seizures	Two or more seizures within 24 h during the same illness
Prolonged seizures	Seizures lasting ≥15 min and documented by a clinician or a nurse
	Convulsing on admission and does not stop after first dose of diazepam
	Use of phenytoin or phenobarbital to stop uncontrolled seizures
	Parental history suggesting of seizures lasting ≥15 min, for example, a child convulsing all way to the hospital for those living more than a kilometer away, seizures lasting more than the period of boiling a pot of maize, milking a dairy cow, or lasting more than the news broadcast on the radio
Focal seizures	Convulsions localized to or starting from one part of the body
	Rolling of eye, cycling movements, or twitching of mouth localized to one body side
	Lateralized asymmetric body weakness postictally (Todd's paralysis)
	Motor deficits of the limbs of one side postseizure event

MAS in cerebral malaria (defined as impaired consciousness [Blantyre Coma Score ≤ 2] in a child with peripheral malaria parasitemia in whom other causes of encephalopathy such as hypoglycemia and bacterial meningitis were excluded) are in this study referred to as “MAS with coma” because the occurrence of prolonged seizures may have contributed to the depressed conscious level in some children.

The genetic associations with MAS were investigated for six phenotypes, namely, all MAS, all focal MAS, all prolonged MAS, all repetitive MAS, all complex MAS, and all MAS with coma. In addition to being common in malaria, complex MAS are thought by some authors to form a more homogeneous group for genetic studies (Baulac et al., 2004).

### Genetic data

Venous blood collected at admission from study subjects was separated by centrifugation, and the packed cells were stored at −80°C prior to subsequent extraction of DNA using commercially available kits according to the manufacturer's instructions. Genomic DNA samples then underwent whole genome amplification through either Primer Extension Pre-amplification (PEP; Zhang et al., 1992) or Multiple Displacement Amplification (MDA; Gonzalez et al., 2005), before genotyping on a Sequenom® Mass Array genotyping platform (Ross et al., 1998). Details of the malaria candidate polymorphisms genotyped, including the frequencies of major alleles (“wild type alleles” that are dominant and most frequent) and minor alleles (“mutant alleles” that are less dominant and rare), are shown in Table S1.

### Statistical analysis

All statistical analysis was performed using STATA (version 11; Stata Corp, College Station, Texas, U.S.A.). Quality control procedures were applied to the genotype data across both individuals and polymorphisms. For example, polymorphisms with >10% genotypes missing duplicated records and individuals with excessive missing genotypes (>10%) were removed. We did not exclude polymorphisms on the basis of Hardy-Weinberg equilibrium deviation because both cases and controls were children admitted to hospital.

Logistic regression models were used to measure polymorphism's association with MAS or complex MAS (odds ratios). The model could not run the associations for pure focal seizures in all sites and pure prolonged seizures in Muheza because of lack of power due to small numbers. Models included the polymorphism of interest assuming several related genotypic mechanisms (additive, dominant, recessive, heterozygous advantage) and general models (mutant homozygous and heterozygous gene against wild homozygous gene). Based on an a priori assumption of potentially confounding effects of country ethnic groups in each site (accounts for between site differences), age, sex, and adverse perinatal events (defined as delay in breathing, crying and breastfeeding after birth) on the genetic susceptibility for seizures in malaria, we adjusted for these factors in the genotypic logistic regression models. We report the minimum p-value from the correlated genotypic tests or models. However, significant models with very large odds ratios (>100) were not reported as this could have introduced sparse-data bias due to infrequent genes in some categories.

Pooled estimates for genotypic tests were computed by performing a logistic regression on combined raw data across the four sites. We chose the raw data method after establishing that its findings were similar to the individual site estimates combined using the meta-analysis method that utilizes both fixed effects (inverse-variance weighting) and random effects models (where heterogeneity across the sites is evident; DerSimonian & Laird, 1986). Meta-analysis was used to compute Cochran's Q statistic for estimating genotypic tests' heterogeneity across the sites.

Because performing multiple statistical tests leads to inflation in the occurrence of false positives, we adjusted for multiple testing using a permutation approach that accounted for correlation between markers and tests (Gao et al., 2008). The permutation approach was preferred to Bonferroni correction because the polymorphisms in this analysis were dependent on each other. A derived p-value of 0.009 was estimated to be significant for all genotypic tests, and was based on both permutation adjustment (Gao et al., 2008) and authors' judgment that it was neither too liberal to include false-positive nor too conservative to exclude true-positive associations (Fig. S1).

All DNA samples were collected and genotyped after approval was provided by the relevant research ethics committees and written informed consent was provided by the participants.

## Results

### The distribution of phenotypes of MAS

**Figure 1:**
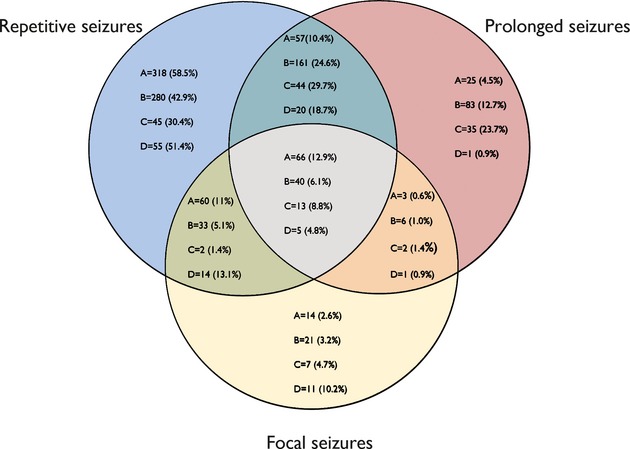
Proportion of complex seizures in each site. A: Blantyre, Malawi (N = 543); B: Kilifi, Kenya (N = 652); C: Kumasi, Ghana (N = 148); D: Muheza, Tanzania (N = 107). Percentages were computed using denominators as numbers for each site.

Of the 2,095 cases of MAS across the sites, and the proportion of complex MAS pooled from across the sites was 70.7%. Blantyre and Kilifi had the highest proportion of complex MAS (543 [70.5%] and 652 [70.4%], respectively; Fig. 1).

Focal MAS occurred in 306 children (14.6%) overall, and the proportion was highest in Blantyre (143 [18.6%]; Fig. 1). Prolonged MAS occurred in 579 children (27.6%) overall, and the proportion was highest (94 [37.6%]) in Kumasi. Repetitive seizures occurred in 1,239 (59.1%) children overall, and the proportion was highest in Blantyre (501 [65.1%]).

The proportion of complex MAS that was focal was highest in Muheza (31 [29.0%]) (Fig. 1). The proportion of complex MAS that was prolonged was highest in Kumasi 94 (63.5%). The proportion of complex MAS that was repetitive was highest in Blantyre 501 (92.3%). There was considerable overlap between the complex phenotypes and the distribution differed across the sites (Fig. 1).

The proportion of cases who met the WHO definition of cerebral malaria (also referred to as “MAS with coma” was 1,267/2,095 [60.5%] for all sites, 713/770 [92.6%] for Blantyre, 410/900 [45.6%] for Kilifi, 73/250 [29.2%] for Kumasi, and 71/175 [40.6%] for Muheza). The proportion of cases with a previous history of seizures was 320/2,095 (15.3%) for all sites, 190/770 (24.7%) for Blantyre, 100/900 (11.1%) for Kilifi, 29/250 (11.6%) for Kumasi, and 1/175 (0.6%) for Moshi.

### Minor allele frequencies and genotypic tests

The minor allele frequencies for the polymorphisms considered in the final analysis are summarized in Table S1. Generally there was little heterogeneity of minor allele frequencies between cases and controls either within or between each site. Following filtering of the single nucleotide polymorphisms (SNPs) as described in Methods, analysis for association with MAS was undertaken using several genetic polymorphisms (Tables 2–7). Results for SNPs where the p-values from the analysis were <0.009 (to allow for multiple testing [see Methods]) were identified and are shown with the data from across all sites.

**Table 2:** Polymorphisms associated with all malaria-associated seizures. (A) Site-specific and (B) pooled analysis across sites

(A)												

	Blantyre, Malawi			Kilifi, Kenya			Kumasi, Ghana			Muheza, Tanzania		
				
Polymorphism	Odds ratio	p-value	Model	Odds ratio	p-value	Model	Odds ratio	p-value	Model	Odds ratio	p-value	Model
IL10 rs1800896	0.37 (0.18–0.74)	0.00531	rec	0.85 (0.62–1.16)	0.29979	rec	0.53 (0.33–0.85)	0.00739	het	1.22 (0.64–2.34)	0.53950	rec
EMR1 rs373533	4.96 (1.78–13.81)	0.00219	add	1.18 (0.92–1.52)	0.19266	gen	0.52 (0.33–0.81)	0.00360	het	0.58 (0.35–0.99)	0.04401	rec
EMR1 rs461645	0.26 (0.10–0.68)	0.00678	add	1.16 (0.94–1.42)	0.15859	het	0.55 (0.36–0.86)	0.00820	het	1.43 (0.87–2.36)	0.16016	dom
G6PD rs1050828 (male)	0.85 (0.44–1.64)	0.63689	rec	0.67 (0.46–0.99)	0.043059	rec	0.26 (0.11–0.60)	0.00155	dom	1.40 (0.33–8.51)	0.51996	gen
G6PD rs1050829 (male)	1.66 (0.93–3.00)	0.08877	gen	0.66 (0.16–2.74)	0.56976	add	0.27 (0.11–0.62)	0.00230	dom	0.58 (0.15–2.26)	0.42957	het
CD40LG rs3092945 (female)	8.07 (1.65–37.25)	0.00953	het	0.43 (0.26–0.69)	0.00068	rec	1.48 (0.82–2.68)	0.19521	dom	0.32 (0.10–1.21)	0.094107	rec
CR1 rs17047660	1.58 (0.87–2.91)	0.13540	het	2.74 (1.44–5.20)	0.00212	rec	0.83 (0.53–1.30)	0.42305	dom	0.68 (0.43–1.06)	0.09166	het
TNF rs3093662	0.68 (0.42–1.09)	0.10847	het	1.46 (1.14–1.88)	0.00274	het	0.54 (0.16–1.87)	0.3359	het	7.22 (0.78–67.13)	0.08220	gen
CR1 rs17047661	1.55 (0.75–3.22)	0.23483	add	1.14 (0.80–1.64)	0.46397	gen	0.49 (0.21–1.17)	0.10869	gen	0.37 (0.19–0.72)	0.00354	gen

(B)												

			Pooled analysis across the four sites									
	
Gene			Odds ratio (95% CI)		p-value							Model
IL10 rs3024500			1.37 (1.09–1.71)		0.00846							gen
IL17RE rs708567			0.78 (0.65–0.94)		0.00709							rec

Genotypic tests with a p-value ≤ 0.009 are reported, with comparisons from other sites (on adjustment for multiple testing, a p-value of ≤0.009 was considered to represent true genotypic tests). The logistic model was adjusted for age, sex, ethnicity, and history of adverse perinatal events. Model denotes the inheritance pattern of interest: add = additive, dom = dominant, het = heterozygous, and rec = recessive. Polymorphisms are represented as HUGO gene symbol and rs number.

**Table 3:** Polymorphisms associations with combined complex malaria-associated seizures. (A) Site specific and (B) pooled analysis across sites

(A)												

	Blantyre, Malawi			Kilifi, Kenya			Kumasi, Ghana			Muheza, Tanzania		
				
Gene	Odds ratio	p-value	Model	Odds ratio	p-value	Model	Odds ratio	p-value	Model	Odds ratio	p-value	Model
IL10 rs1800896	0.43 (0.20–0.92)	0.02894	rec	0.77 (0.53–1.12)	0.17754	gen	0.43 (0.24–0.75)	0.00309	het	1.29 (0.55–3.01)	0.56062	gen
IL4 rs2243250	0.55 (0.18–0.73)	0.00960	rec	1.10 (0.86–1.40)	0.43910	het	0.86 (0.50–1.49)	0.61018	het	0.87 (0.51–1.49)	0.61917	het
EMR1 rs373533	4.43 (1.47–13.34)	0.00813	het	1.23 (0.97–1.55)	0.08112	het	0.38 (0.23–0.65)	0.00033	het	1.40 (0.84–2.33)	0.193088	het
EMR1 rs461645	0.27 (0.10–0.80)	0.01831	gen	1.27 (1.02–1.61)	0.03680	het	0.41 (0.25–0.68)	0.00055	het	1.36 (0.82–2.24)	0.22393	het
CR1 rs17047660	0.24 (0.03–2.09)	0.19451	gen	2.94 (1.49–5.80)	0.00183	rec	0.83 (0.49–1.42)	0.49958	het	1.85 (0.59–5.78)	0.28809	rec
CTL4 rs2242665	1.29 (0.65–2.56)	0.46678	rec	1.38 (0.87–2.19)	0.16543	gen	0.61 (0.25–1.53)	0.29522	het	1.98 (1.19–3.30)	0.00833	het
ICAM1 rs5498	1.90 (1.17–3.08)	0.00902	het	0.69 (0.51–0.93)	0.015872	gen	0.73 (0.38–1.41)	0.35340	het	1.16 (0.66–2.04)	0.60455	dom
IL17RE rs708567	0.70 (0.41–1.21)	0.205212	rec	0.65 (0.47–0.90)	0.00889	add	0.69 (0.36–1.36)	0.29232	rec	1.69 (0.78–3.68)	0.18357	gen

(B)												

			Pooled analysis across the four sites									
	
Gene			Odds ratio (95% CI)		p-value							Model
–			–		–							–

Genotypic tests with a p-value ≤ 0.009 are reported, with comparisons from other sites (on adjustment for multiple testing, a p-value of ≤0.009 was considered to represent true genotypic tests). The logistic model was adjusted for age, sex, ethnicity, and history of adverse perinatal events. Model denotes the inheritance pattern of interest: add = additive, dom = dominant, het = heterozygous, and rec = recessive. The cells with missing data (–) represents genotypic tests, which did not reach significant levels in the pooled analysis. Polymorphisms are represented as HUGO gene symbol and rs number.

**Table 4:** Polymorphisms associations with focal malaria-associated seizures. (A) Site-specific and (B) pooled analysis across sites

(A)												

	Blantyre, Malawi			Kilifi, Kenya			Kumasi, Ghana			Muheza, Tanzania		
				
Polymorphism	Odds ratio	p-value	Model	Odds ratio	p-value	Model	Odds ratio	p-value	Model	Odds ratio	p-value	Model
IL20RA rs1555498	0.50 (0.15–1.70)	0.27119	add	0.93 (0.48–1.82)	0.83213	gen	0.26 (0.03–2.17)	0.211485	add	3.86 (1.67–8.93)	0.00168	rec
CD36 rs3211938	0.65 (0.17–2.44)	0.524159	dom	1.47 (0.81–2.70)	0.20875	het	10.16 (1.79–57.83)	0.00894	het	1.53 (0.56–4.22)	0.402252	het

(B)												

			Pooled analysis across the four sites									
	
Gene			Odds ratio (95% CI)		p-value							Model
–			–		–							–

Genotypic tests with a p-value ≤ 0.009 are reported, with comparisons from other sites (on adjustment for multiple testing, a p-value of ≤0.009 was considered to represent true genotypic tests). The logistic model was adjusted for age, sex, ethnicity, and history of adverse perinatal events. Model denotes the inheritance pattern of interest: add = additive, dom = dominant, het = heterozygous, and rec = recessive. The cells with missing data (–) represents genotypic tests, which did not reach significant levels in the pooled analysis. Polymorphisms are represented as HUGO gene symbol and rs number.

**Table 5:** Polymorphisms associations with prolonged malaria-associated seizures. (A) Site-specific and (B) pooled analysis across sites

(A)												

	Blantyre, Malawi			Kilifi, Kenya			Kumasi, Ghana			Muheza, Tanzania		
				
Polymorphism	Odds ratio	p-value	Model	Odds ratio	p-value	Model	Odds ratio	p-value	Model	Odds ratio	p-value	Model
EMR1373533	2.32 (0.57–9.42)	0.23843	het	1.44 (1.05–1.99)	0.02510	het	0.41 (0.23–0.74)	0.00033	het	0.19 (0.04–0.95)	0.043112	add
EMR1 rs461645	2.61 (0.66–10.33)	0.17128	het	1.50 (1.09–2.07)	0.01254	het	0.40 (0.28–0.7)	0.00147	het	2.88 (0.54–18.36)	0.20131	add
CR1 rs17047660	0.39 (0.10–1.62)	0.19522	gen	3.92 (1.71–8.99)	0.00121	gen	1.51 (0.52–4.42)	0.44375	rec	3.15 (0.54–18.36)	0.20131	gen
IL17RE rs708567	1.24 (0.49–3.12)	0.64466	gen	0.55 (0.35–0.86)	0.00917	add	1.30 (0.74–2.32)	0.35336	het	2.66 (0.59–12.01)	0.201649	dom

(B)												

				Pooled analysis across the four sites								
	
Gene				Odds ratio (95% CI)	p-value							Model
CR1 rs17047660				2.56 (1.41–4.67)	0.00194							rec
G6PD rs1050828				0.45 (0.27–0.74)	0.00155							dom

Genotypic tests with a p-value ≤ 0.009 are reported, with comparisons from other sites (on adjustment for multiple testing, a p-value of ≤0.009 was considered to represent true genotypic tests). The logistic model was adjusted for age, sex, ethnicity, and history of adverse perinatal events. Model denotes the inheritance pattern of interest: add = additive, dom = dominant, het = heterozygous, and rec = recessive. Polymorphisms are represented as HUGO gene symbol and rs number.

**Table 6:** Polymorphisms associated with repetitive malaria-associated seizures. (A) Site specific and (B) pooled analysis across sites

(A)												

	Blantyre, Malawi			Kilifi, Kenya			Kumasi, Ghana			Muheza, Tanzania		
				
Polymorphism	Odds ratio	p-value	Model	Odds ratio	p-value	Model	Odds ratio	p-value	Model	Odds ratio	p-value	Model
CR1 rs17047660	0.27 (0.03–3.49)	0.25132	gen	3.04 (1.51–6.12)	0.00180	rec	2.17 (0.70–6.75)	0.17981	gen	2.24 (0.68–7.33)	0.18336	gen
IL4 rs2243250	0.29 (0.11–0.73)	0.00884	gen	1.12 (0.87–1.40)	0.37876	het	0.66 (0.18–2.43)	0.53585	gen	0.71 (0.40–1.26)	0.24413	het
EMR1 rs373533	5.74 (1.67–19.6)	0.00545	gen	1.27 (0.94–1.72)	0.11501	gen	0.27 (0.14–0.50)	0.00003	het	0.74 (0.39–1.40)	0.35779	rec
EMR1 rs461645	0.23 (0.07–0.73)	0.01243	gen	1.21 (0.95–1.55)	0.121345	het	0.38 (0.21–0.69)	0.00127	het	1.24 (0.65–2.37)	0.52182	add
ICAM1 rs5498	1.97 (1.21–3.21)	0.00676	het	0.70 (0.51–0.97)	0.03135	gen	0.60 (0.27–1.34)	0.20767	het	1.11 (0.61–2.01)	0.73366	add
CD40LG rs3092945 (female)	10.90 (1.92–62.05)	0.00706	gen	0.49 (0.28–0.86)	0.012395	rec	1.06 (0.38–2.95)	0.90875	het	0.61 (0.16–2.42)	0.48705	add
IL17RE rs708567	0.65 (0.37–1.15)	0.13993	rec	0.64 (0.46–0.90)	0.00951	rec	1.10 (0.62–1.97)	0.72768	het	1.63 (0.73–3.69)	0.23548	gen
ABO rs8176746	0.74 (0.46–1.19)	0.21816	dom	2.27 (1.24–4.17)	0.00803	rec	0.69 (0.38–1.24)	0.21205	het	0.25 (0.03–2.03)	0.19634	rec
IL10 rs1800896	1.62 (1.03–2.55)	0.03673	het	0.81 (0.55–1.20)	0.30182	gen	0.42 (0.22–0.81)	0.00934	het	1.54 (0.65–3.65)	0.32719	gen

(B)												

			Pooled analysis across the four sites									
	
Gene			Odds ratio (95% CI)		p-value							Model
CR1 rs17047660			1.97 (1.22–3.22)		0.00547							rec
CFTR rs171140229			0.60 (0.41–0.86)		0.00543							het
G6PD rs1050829 (males)			0.29 (0.11–0.72)		0.00817							gen

Genotypic tests with a p-value ≤ 0.009 are reported, with comparisons from other sites (on adjustment for multiple testing, a p-value of ≤0.009 was considered to represent true genotypic tests). The logistic model was adjusted for age, sex, ethnicity, and history of adverse perinatal events. Model denotes the inheritance pattern of interest: add = additive, dom = dominant, het = heterozygous, and rec = recessive. Polymorphisms are represented as HUGO gene symbol and rs number.

**Table 7:** Polymorphisms associated with malaria-associated seizures with coma. (A) Site specific and (B) pooled analysis across sites

(A)												

	Blantyre, Malawi			Kilifi, Kenya			Kumasi, Ghana			Muheza, Tanzania		
				
Polymorphism	Odds ratio	p-value	Model	Odds ratio	p-value	Model	Odds ratio	p-value	Model	Odds ratio	p-value	Model
IL10 rs1800896	0.35 (0.17–0.73)	0.00475	rec	0.71 (0.45–1.13)	0.14675	rec	0.39 (0.18–0.87)	0.02062	gen	1.17 (0.65–2.13)	0.60259	dom
EMR1 rs373533	4.58 (1.62–19.6)	0.00405	add	1.29 (0.97–1.72)	0.07953	het	0.26 (0.13–0.53)	0.00019	het	0.43 (0.18–1.03)	0.05695	add
EMR1rs461645	0.26 (0.10–0.73)	0.01021	gen	1.35 (1.02–1.80)	0.03569	het	2.95 (1.49–5.90)	0.00198	rec	2.01 (0.87–4.65)	0.10129	add
CD40LG rs1126535 (female)	0.41 (0.21–0.81)	0.00988	het	1.43 (0.93–2.20)	0.10370	dom	1.96 (0.34–11.5)	0.45469	het	3.53 (1.42–8.79)	0.00659	het
CD40LG rs3092945 (female)	8.27 (1.74–39.21)	0.00781	rec	0.62 (0.35–1.09)	0.10165	rec	0.65 (0.16–2.74)	0.56353	gen	0.26 (0.10–2.13)	0.21076	rec
CR1rs17047660	1.81 (0.96–3.42)	0.06569	het	3.47 (1.51–7.99)	0.00341	gen	1.17 (0.59–2.37)	0.64389	het	0.25 (0.11–0.60)	0.00177	add

(B)												

			Pooled analysis across the four sites									
	
Gene			Odds ratio (95% CI)		p-value							Model
IL13 rs20541			0.72 (0.52–0.91)		0.00601							dom
IL10 rs3024500			1.55 (1.21–2.00)		0.00064							dom

Genotypic tests with a p-value ≤ 0.009 are reported, with comparisons from other sites (on adjustment for multiple testing, a p-value of ≤0.009 was considered to represent true genotypic tests). The logistic model was adjusted for age, sex, ethnicity, and adverse perinatal events. Model denotes the inheritance pattern of interest: add = additive, dom = dominant, het = heterozygous, and rec = recessive. The cells with missing data (–) represents assays that had missing data or poor fidelity. Polymorphisms are represented as HUGO gene symbol and rs number.

### Polymorphisms associated with all MAS

All-MAS encompasses all MAS phenotypes and two genes were significantly associated with all-MAS in at least two sites, IL10-rs1800896 in Blantyre (odds ratio [OR] = 0.37 [95% confidence interval (CI), 0.18–0.74], p = 0.00531, recessive model) and Kumasi (OR = 0.53 [95% CI, 0.33–0.84], p = 0.00739, heterozygous model) and epidermal growth factor module-containing mucin-like hormone receptor (EMR)1-rs373533 in Blantyre (4.96 [95% CI, 1.78–13.81], p = 0.00219, additive model) and Kumasi (OR = 0.52 [95% CI, 0.33–0.81], p = 0.00360, heterozygous model). A second SNP (rs461645) in EMR1 was also significant in Blantyre and Kumasi due to high linkage disequilibrium with EMR1-rs373533 in all the ethnic groups (r^2^ > 0.9, data not shown). Others shown in Table 2 were significantly associated with MAS in one site only.

Most polymorphisms were not significant in analysis pooled across-site analysis except for IL10-rs3024500 (OR = 1.36 [95% CI, 1.08–1.71], p = 0.00651, general model) and IL-17 receptor E-rs708567 (OR = 0.78 [95% CI, 0.65–0.94], p = 0.00709, recessive model). We also looked for associations in the sub-phenotypes of MAS to reduce heterogeneity of the phenotype, reported below, although there would be some loss of power in the sample sizes. Meta-analysis of association estimates of IL10-rs1800896 in the all MAS phenotype showed that there was considerable heterogeneity across the sites (68.7% [95% CI 34.8–93.3%]).

### Polymorphisms associated with all-complex MAS

This phenotype comprises the prolonged, focal, and repetitive phenotypes that together define the majority of the malaria-associated seizures. We analyzed them together, since they have some degree of overlap (Fig. 1). Several genes showed a significant association in at least one site (Table 3), two of which were significant in all-MAS above, namely IL10-rs1800896 (OR = 0.43 [95% CI, 0.24–0.75], p = 0.00309, heterozygous model) in Kumasi and EMR1-rs373533 in Blantyre (OR = 4.43 [95% CI, 1.47–13.34], p = 0.00813, heterozygous model) and Kumasi (OR = 0.38 [95% CI, 0.23–0.65], p = 0.00033, heterozygous model). None of these SNPs were associated with complex MAS in a pooled analysis.

### Polymorphisms associated with focal-MAS, prolonged-MAS, and repetitive-MAS

Several polymorphisms, none of which appeared in MAS and complex MAS, were significantly associated with focal MAS, particularly IL-20 receptor A-rs1555498 (OR = 3.86 [95% CI, 1.67–8.97], p = 0.00158, recessive model) in Moshi and CD36-rs3211938 (OR = 10.16 [95% CI, 1.79–57.83], p = 0.00894, recessive model) for Kumasi. However, there was no significant association with focal MAS in a pooled analysis across the sites.

Three polymorphisms that appeared significant in MAS and/or complex MAS were associated with prolonged MAS (Tables 3 and 4), EMR1-rs461645 (OR = 0.41 [95% CI, 0.23–0.74], p = 0.00336, heterozygous model) in Kumasi and CR1-rs1704660 (OR = 3.92 [95% CI, 1.71–8.99], p = 0.00121, general model) and IL17RE-rs708567 (OR = 0.55 [95% CI, 0.35–0.86], p = 0.00917, additive model) in Kilifi. Only CRI-rs1704660 from these two polymorphisms was significant in a pooled analysis across the sites (Table 5).

Two polymorphisms identified as significant in MAS, complex MAS, or prolonged MAS (Tables 3 and 4), were associated with repetitive MAS, EMRI-rs373533 in Blantyre (OR = 5.73 [95% CI, 1.67–19.65], p = 0.00545, general model) and Kumasi (OR = 0.27 [95% CI, 0.14–0.50], p = 0.00003, heterozygous model) and CR1-rs17047660 (OR = 3.04 [95% CI, 1.51–6.12], p = 0.00180, recessive model) in Kilifi. Of these two polymorphisms only CR1-rs17047660 (OR = 1.97 [95% CI, 1.22–3.22], p = 0.00547, recessive model) was associated with repetitive MAS in the pooled analysis, and others were as well (Table 6).

### Polymorphisms association with MAS with coma/cerebral malaria

Two polymorphisms associated with most of the above phenotypes (Tables 2–6) were also significant for MAS with coma, EMRI-rs373533 in Blantyre (OR = 4.58 [95% CI, 1.62–12.93], p = 0.00405, additive model) and Kumasi (OR = 0.26 [95% CI, 0.13–0.53], p = 0.00019, dominant model) and CR1-rs17047660 in Kilifi (OR = 3.47 [95% CI, 1.51–7.99], p = 0.00341, general model), Kilifi and Moshi (OR = 0.25 [95% CI, 0.11–0.60], p = 0.00177, additive model). CD40-rs1126935 too was associated with MAS with coma in more than one site (Table 7).

## Discussion

This is the first study to investigate the effect of candidate genes for malaria infection on the risk of acute seizures across multiple sites in Africa. Using established definitions for seizure phenotypes (Table 1), our data show that a significant proportion (>70%) of children admitted to hospitals in sub-Saharan Africa with severe malaria exhibit seizures with complex features (prolonged, focal, or repetitive), but the proportions of the different phenotypes vary between sites. A number of different polymorphisms (including the X-chromosome) were associated with the risk of the main phenotypes of MAS at each site, and in a pooled analysis. Polymorphisms of four genes were identified as significant in most of the subgroups and sites (CR1-rs1704660, IL10-rs1800896, and EMR1-rs373533/rs461645 and CD40-rs3092945 [females]), although only CR1 showed an overall significant association across the sites in the prolonged and repetitive MAS phenotypes. Other polymorphisms did show positive association but mostly for one phenotype and site (Table 3). Of the positive association polymorphisms several are thought to code for molecules that could have a putative role in the pathogenesis of seizures in falciparum malaria.

The classification of seizures is largely based on phenotypes found in febrile seizures (Baram & Shinnar, 2002), but some cases may have been misclassified, since generalized seizures may have had a focal origin with rapid secondary generalization (Gwer et al., 2012). We were not able to determine relatedness of the study participants, which may affect the associations, but adjusting for ethnicity did in part account for this. Electroencephalography and neuroimaging were not routinely performed at the time of the study due to logistic reasons. The strength of our study lies in the large sample size from combining studies across four countries, which allowed us to use a control group that comprised the severe malaria cases without seizures, thus ensuring that any associations identified represent the risk of developing acute seizures in malaria. The genotypic tests p-values are reported as they were generated from the analyzing software, so are easier to interpret.

Polymorphisms demonstrated heterogeneities in associations with MAS across the sites. These heterogeneities may be ascribed to sample size differences and the resulting power (Muheza had the smallest samples), possible differences in documenting the three phenotypes of complex MAS, haplotypic (polymorphisms on one or several loci that may be associated) differences between populations, and/or the effects of population structure. We tried to minimize the latter by adjusting for ethnicity within sites. Some of the heterogeneous associations in the sites may be due to linkage disequilibrium. Because we adjusted for multiple testing, such site-specific associations could be due to strong linkage disequilibrium with actual causal polymorphisms not investigated in this study. The finding that a gene was protective in one site and increased risk in another can be explained by the different inheritance models in the affected sites, or could suggest that the associations are related to a gene region rather than the effect of the particular polymorphism.

In these associations, we studied polymorphisms that were genotyped as part of MalariaGEN consortium project (MalariaGEN, 2008), and could not investigate some polymorphisms associated with febrile seizures such as the six susceptibility febrile seizure loci (FEB)1–6, voltage-gated sodium channel genes (*SCN1A*, *SCN1B*, or *SCN2A*), γ-amino-butyric acids genes (GABA(A) or GABRG2) (Nakayama & Arinami, 2006). We did, however, include polymorphisms of the interleukin family (IL-1A, 1L-1B, 1L-4, 1L-20RA), some of which have been previously investigated in febrile seizures studies (Tsai, 1987; Virta et al., 2002; Kira et al., 2005; Ishizaki et al., 2009).

Some polymorphisms had stronger associations with acute seizures with complex phenotypes, suggesting that these three phenotypes may provide insights into the epileptogenesis of malaria. The finding that specific polymorphisms were replicated across the different complex phenotypes supports the role of these genes in the epileptogenesis of malaria. The complex phenotypes are particularly associated with malaria (Kariuki et al., 2011) and are also important risk factors for development of epilepsy following severe malaria (Birbeck et al., 2010). Those polymorphisms that are found in more than one site are likely to be linked to genes that may play a significant role in the pathogenesis of acute seizures in these children.

The biologic functions of some polymorphisms have not been fully investigated, and can only be speculated. EMR1 is sometimes elevated in placental malaria (Muehlenbachs et al., 2007; Sevastianova et al., 2008), which is characterized by sequestration of parasites, similar to that found in the brain. EMR1 is a membrane receptor that also activates G protein receptor subunit alpha (GNAS) (Auburn et al., 2008), whose subclass C receptors bind glutamate and GABA, the two important neurotransmitters of epileptogenesis. GNAS did not reach the 0.009 significance level in this study but reached the 0.05 level. In addition, EMR1 is the human homologue of F4/80 in mice that defines macrophages, whose elevation in malaria infection precedes leucocyte sequestration into the brain (Wozencraft et al., 1984).

Many of the polymorphisms genotyped in this study have previous published associations with severe malaria, and we have analyzed them in the context of association with seizures. CR1 is postulated to be involved in the adhesion of *P. falciparum* infected erythrocytes to uninfected erythrocytes (rosetting; Nagayasu et al., 2001; Idro et al., 2005) and is therefore thought to play a role in the pathogenesis of seizures in severe malaria. Polymorphisms of the ABO blood system are associated with rosetting of erythrocytes among people of blood types A and B (Cserti & Dzik, 2007), resulting in impaired tissue perfusion that could increase the risk for seizures. Similarly, the association between ICAM1 polymorphisms seizures with severe malaria (Kun et al., 1999) could be explained by the binding of ICAM1 to *P. falciparum*–infected erythrocytes onto brain microvasculature (Berendt et al., 1992; Kun et al., 1999) causing sequestration. CD36 facilitates this sequestration (Cserti & Dzik, 2007). Interleukins are usually increased in falciparum malaria infections, and may directly precipitate seizures because they are inflammatory and pyrogenic in nature (Haysa et al., 1999; Chaisavaneeyakorn et al., 2003; Wilson et al., 2005). G6PD enhances parasite clearance by phagocytosis, a process that can culminate in release of inflammatory molecules often associated with seizures (Sabeti et al., 2002; Wilson et al., 2005). Activation of microglial CD40 results in inflammation-induced seizures (Sun et al., 2008) and underpins the association between this polymorphism and MAS in coma/cerebral malaria.

Our findings have demonstrated that children admitted with falciparum malaria may have a genetic predisposition to acute seizures, but the polymorphisms associated with seizures in malaria vary with the phenotype and sites across Africa. These polymorphisms were particularly associated with the three complex phenotypes of MAS, which could also be important in epileptogenesis of severe malaria. Studies from other malaria-endemic settings are required to confirm these findings, and genome-wide association studies are required to study if other seizures-specific genes determine the risk of acute seizures.

## Supporting Information

Figure S1 The cut-off for significant genotypic tests based on both permutation tests and authors' judgment as both not too conservative and too liberal.

**Table S1.** Minor allele frequencies (MAF) and Hardy-Weinberg equilibrium (HWE) test in the controls for polymorphisms included in the final analysis. **Table S2.** Details of malaria transmission in the study areas across the four sites in Africa.
