# Supplementary figures and images for: The genetic risk of acute seizures in African children with falciparum malaria
Source: Epilepsia. 2013 Apr 24;54(6):990–1001. doi: 10.1111/epi.12173 (PMC3734649; doi:10.1111/epi.12173)

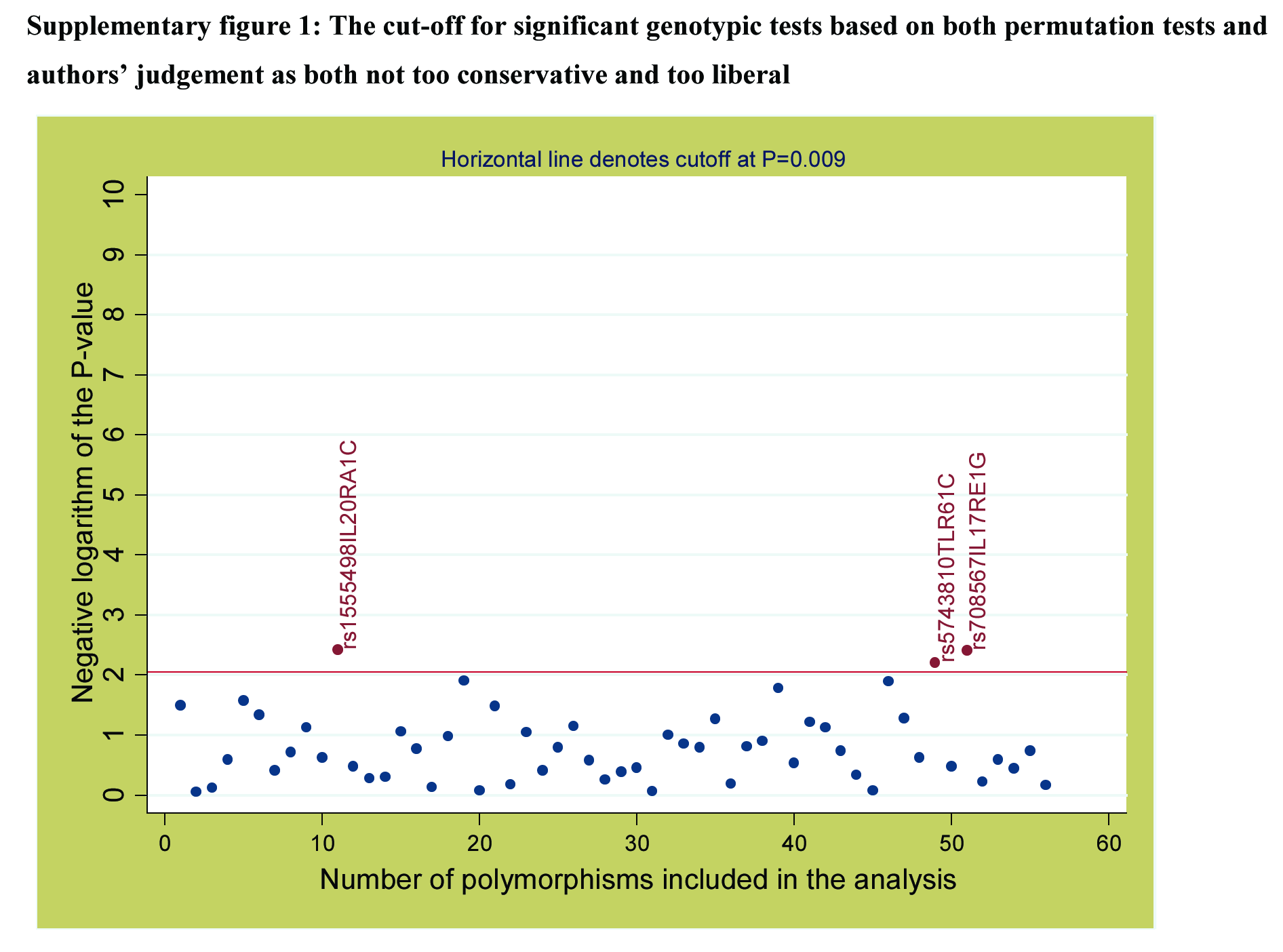

Supplement: Figure S1 — The cut-off for significant genotypic tests based on both permutation tests and authors' judgment as both not too conservative and too liberal. [file epi0054-0990-sd1.tif]
