# Supplementary material for: The genetic risk of acute seizures in African children with falciparum malaria
Source: Epilepsia. 2013 Apr 24;54(6):990–1001. doi: 10.1111/epi.12173 (PMC3734649; doi:10.1111/epi.12173)
Supplement: Supplementary file 2 [file epi0054-0990-sd2.docx]

**Supplementary table 1: Minor allele frequencies (MAF) and Hardy Weinberg Equilibrium (HWE) test in the controls for polymorphisms included in the final analysis.**

|  |  |  | Blantyre, Malawi | | | Kilifi, Kenya | | | Kumasi, Ghana | | | Muheza, Tanzania | | |
| --- | --- | --- | --- | --- | --- | --- | --- | --- | --- | --- | --- | --- | --- | --- |
| Polymorphism | Major allele | Minor allele | cases | controls | HWE  (p-value) | cases | controls | HWE  (p-value) | cases | controls | HWE  (p-value) | cases | controls | HWE  (p-value) |
| GBP7 rs1803632^c^ | G | C | 0.47 | 0.45 | 0.058 | 0.50 | 0.52 | 0.194 | 0.47 | 0.50 | 0.006 | 0.48 | 0.50 | 0.707 |
| FCGR2a rs1801274^a^ | T | C | 0.60 | 0.59 | 0.126 | - | - | - | - | - | - | - | - | - |
| IL10 rs3024500^a^ | G | A | 0.53 | 0.51 | 0.402 | 0.48 | 0.45 | 0.318 | 0.66 | 0.65 | 0.782 | 0.54 | 0.50 | 0.161 |
| IL10 rs1800896^a^ | T | C | 0.32 | 0.31 | 0.036 | 0.35 | 0.36 | 0.757 | 0.26 | 0.27 | 0.211 | 0.30 | 0.30 | 0.952 |
| IL10 rs1800890_a_ | A | T | 0.22 | 0.20 | 0.494 | 0.21 | 0.23 | 0.123 | 0.18 | 0.20 | 0.432 | 0.21 | 0.21 | 0.278 |
| CR1 rs17047660^b^ | A | G | 0.24 | 0.25 | 0.026 | 0.17 | 0.16 | 0.074 | 0.25 | 0.27 | 0.303 | 0.19 | 0.20 | 0.33 |
| CR1 rs17047661^b^ | A | G | 0.69 | 0.68 | 0.662 | 0.66 | 0.66 | 0.986 | 0.72 | 0.76 | 0.685 | 0.58 | 0.67 | 0.744 |
| IL1A rs17561^a^ | G | T | 0.16 | 0.13 | 0.118 | 0.18 | 0.18 | 0.005 | 0.17 | 0.20 | 0.101 | 0.15 | 0.21 | 0.703 |
| IL1A rs1800587^a^ | C | T | 0.36 | 0.35 | 0.097 | m | m |  | m | m |  | m | m |  |
| IL1B rs1143634^a^ | C | T | 0.11 | 0.10 | 0.213 | 0.12 | 0.12 | 0.936 | 0.14 | 0.10 | 0.536 | 0.09 | 0.11 | 0.710 |
| IL17RE rs708567^a^ | G | A | 0.45 | 0.44 | 0.081 | 0.48 | 0.51 | 0.646 | 0.45 | 0.47 | 0.468 | 0.56 | 0.58 | 0.198 |
| TLR9 rs187084^a^ | C | T | 0.72 | 0.75 | 0.070 | 0.68 | 0.66 | 0.829 | 0.73 | 0.65 | 0.174 | 0.68 | 0.66 | 0.234 |
| IL17RD rs6780995^a^ | G | A | 0.58 | 0.57 | 0.686 | 0.61 | 0.59 | 0.066 | 0.57 | 0.51 | 0.661 | 0.54 | 0.60 | 0.700 |
| TLR10 rs11096957 | A | C | 0.59 | 0.57 | 0.208 | m | m | m | - | - | - | - | - | - |
| TLR1 rs4833095^a^ | C | T | 0.08 | 0.07 | 0.124 | 0.09 | 0.09 | 0.703 | 0.12 | 0.11 | 0.327 | 0.08 | 0.08 | 0.044 |
| TLR6 rs5743809^a^ | T | C | 0.07 | 0.08 | 0.041 | 0.07 | 0.06 | 0.441 | 0.04 | 0.04 | 0.186 | 0.10 | 0.07 | 0.821 |
| C6 rs1801033^a^ | A | C | 0.43 | 0.45 | 0.721 | 0.46 | 0.47 | 0.927 | 0.56 | 0.53 | 0.959 | 0.44 | 0.45 | 0.764 |
| IRF1 rs2706384^a^ | C | A | 0.37 | 0.36 | 0.879 | 0.37 | 0.35 | 0.389 | 0.40 | 0.45 | 0.002 | 0.40 | 0.39 | 0.033 |
| IL13 rs20541^a^ | C | T | 0.20 | 0.24 | 0.160 | 0.21 | 0.21 | 0.906 | 0.15 | 0.18 | 0.733 | 0.26 | 0.25 | 0.293 |
| IL4 rs2243250^a^ | C | T | 0.74 | 0.79 | 0.056 | 0.78 | 0.78 | 0.187 | 0.73 | 0.74 | 0.041 | 0.79 | 0.77 | 0.728 |
| LTA rs2239704^a^ | G | T | 0.16 | 0.16 | 0.716 | 0.12 | 0.13 | 0.536 | 0.33 | 0.29 | 0.050 | 0.19 | 0.16 | 0.716 |
| LTA rs909253^a^ | T | C | 0.50 | 0.46 | 0.330 | 0.48 | 0.48 | 0.121 | 0.50 | 0.54 | 0.288 | 0.47 | 0.52 | 0.394 |
| TNF rs1799964^a^ | T | C | 0.20 | 0.25 | 0.106 | 0.28 | 0.27 | 0.172 | 0.13 | 0.14 | 0.261 | 0.24 | 0.24 | 0.863 |
| TNF rs1800629^a^ | G | A | 0.11 | 0.09 | 0.525 | 0.08 | 0.09 | 0.228 | 0.09 | 0.12 | 0.801 | 0.11 | 0.09 | 0.265 |
| TNF rs3093662^a^ | A | G | 0.11 | 0.14 | 0.110 | 0.13 | 0.10 | 0.173 | - | - | - | 0.12 | 0.10 | 0.156 |
| CTL4 rs2242665^a^ | A | G | 0.73 | 0.71 | 0.831 | 0.75 | 0.72 | 0.013 | 0.70 | 0.72 | 0.464 | 0.74 | 0.79 | 0.728 |
| IL20RA1 rs1555498^a^ | C | T | 0.50 | 0.45 | 0.941 | 0.48 | 0.48 | 0.167 | 0.36 | 0.40 | 0.435 | 0.51 | 0.48 | 0.291 |
| NOD1 rs2075820^a^ | G | A | 0.40 | 0.37 | 0.04 | 0.37 | 0.37 | 0.45 | 0.41 | 0.40 | 0.015 | 0.37 | 0.40 | 0.205 |
| CD36 rs3211938^b^ | T | G | 0.08 | 0.07 | 0.771 | 0.09 | 0.09 | 0.054 | 0.09 | 0.08 | 0.034 | 0.10 | 0.08 | 0.710 |
| CFTR rs17140229^c^ | T | C | 0.29 | 0.28 | 0.295 | 0.31 | 0.33 | 0.757 | 0.42 | 0.43 | 0.442 | 0.28 | 0.28 | 0.105 |
| TLR4 rs4986790^a^ | A | G | - | 0.04 | 0.036 | - | - | - | 0.10 | 0.12 | 0.037 | - | 0.06 | 0.578 |
| TLR4 rs4986791^a^ | C | T | - | - | - | - | - | - | - | - | - | - | 0.01 | <0.001 |
| ABO rs8176746^c^ | C | A | 0.17 | 0.18 | 0.841 | 0.17 | 0.16 | 0.944 | 0.24 | 0.26 | 0.014 | 0.15 | 0.17 | 0.116 |
| HBB1 rs334^c^ | A | T | 0.01 | - | - | - | 0.02 | <0.001 | - | 0.04 | <0.001 | - | 0.02 | <0.001 |
| NOS2 rs2297518^a^ | G | A | 0.11 | 0.14 | 0.601 | 0.13 | 0.13 | 0.006 | 0.06 | 0.06 | 0.280 | 0.15 | 0.14 | 0.787 |
| NOS2 rs1800482^a^ | G | C | 0.08 | 0.08 | 0.745 | 0.08 | 0.07 | 0.066 | 0.09 | 0.13 | 0.955 | - | - | - |
| NOS2 rs9282799^a^ | C | T | 0.06 | 0.05 | 0.645 | - | - | - | - | - | - | - | - | - |
| NOS2 rs8078340^a^ | C | T | 0.24 | 0.27 | 0.250 | 0.19 | 0.20 | 0.758 | 0.20 | 0.16 | 0.565 | 0.22 | 0.24 | 0.773 |
| EMR1 rs373533^b^ | G | T | 0.55 | 0.51 | 0.454 | 0.50 | 0.47 | 0.426 | 0.41 | 0.44 | 0.0008 | 0.42 | 0.49 | 0.235 |
| EMR1 rs461645^b^ | T | C | 0.44 | 0.47 | 0.608 | 0.49 | 0.52 | 0.129 | 0.56 | 0.52 | 0.0008 | 0.56 | 0.50 | 0.280 |
| ICAM1 rs5491^b^ | A | T | 0.28 | 0.27 | 0.552 | 0.40 | 0.30 | 0.130 | - | - | - | - | - | - |
| ICAM1 rs5498^b^ | A | G | 0.14 | 0.15 | 0.530 | 0.10 | 0.11 | 0.224 | 0.11 | 0.12 | 0.912 | 0.12 | 0.12 | 0.034 |
| GNAS rs2057291^a^ | G | A | 0.23 | 0.25 | 0.568 | 0.19 | 0.22 | 0.829 | - | - | - | - | - | - |
| GNAS rs8386^a^ | C | T | 0.13 | 0.13 | 0.628 | 0.19 | 0.18 | 0.398 | 0.19 | 0.24 | 0.935 | 0.17 | 0.16 | 0.385 |
| DERL3 rs1128127^c^ | G | A | 0.47 | 0.46 | 0.274 | 0.46 | 0.46 | 0.928 | 0.50 | 0.51 | 0.402 | 0.49 | 0.50 | 0.478 |
| CD40LG rs1126535 (female)^a^ | T | C | 0.19 | 0.21 | 0.333 | 0.15 | 0.16 | <0.001 | 0.011 | 0.12 | <0.001 | 0.17 | 0.17 | <0.001 |
| CD40LG rs1126535 (male)^a^ | T | C | 0.19 | 0.16 | <0.001 | 0.14 | 17 | <0.001 | 0.10 | 0.08 | 0.062 | 0.20 | 0.26 | <0.001 |
| CD40LG rs3092945 (female)^a^ | T | C | 0.21 | 0.23 | 0.497 | 0.24 | 0.24 | <0.001 | 0.39 | 0.33 | <0.001 | 0.25 | 0.26 | 0.163 |
| CD40LG rs3092945 (male)^a^ | T | C | 0.23 | 0.24 | <0.001 | 0.28 | 28 | <0.001 | 0.35 | 0.26 | 0.183 | 0.24 | 0.20 | <0.001 |
| G6PD rs1050828 (female)^c^ | C | T | 0.17 | 0.21 | 0.504 | 0.19 | 0.23 | <0.001 | 0.18 | 19 | <0.001 | 0.18 | 0.17 | <0.001 |
| G6PD rs1050828 (male)^c^ | C | T | 0.19 | 0.23 | <0.001 | 0.18 | 23 | <0.001 | 0.13 | 0.26 | 0.835 | 0.14 | 0.15 | <0.001 |
| G6PD rs1050829 (female)^c^ | T | C | 0.36 | 0.42 | 0.390 | 0.39 | 0.42 | <0.001 | - | 0.39 | <0.001 | 0.34 | 0.34 | 0.028 |
| G6PD rs1050829 (male)^c^ | T | C | 0.41 | 0.42 | <0.001 | 0.41 | 0.42 | <0.001 | 0.39 | 0.54 | 0.875 | 0.38 | 0.35 | <0.001 |
| Polymorphisms are represented as the HUGO gene symbol followed by the polymorphism rs number and sorted into chromosomal order  Cells with missing data (-) represent assays that had missing data or poor fidelity; (m) represents polymorphisms that were monomorphic. The superscripts alphabets after the gene names denote the biological selection criteria for which a=inflammation, b=sequestration/adhesion and c=impaired oxygenation/neurotransmission | | | | | | | | | | | | | | |

**Supplementary Table 2: Details of malaria transmission in the study areas across the four sites in Africa**

| **Kumasi, Ghana** |
| --- |
| Studies were conducted in the Kumasi Metropolitan District of the Ashanti Region of Ghana, West Africa which is at an altitude of about 250-300m in a transitional forest zone within the wet sub-equatorial climate zone. Temperature range between 21.5^o^C and 30.7^o^C with the daily humidity changing from about 84% in the morning to 60% in the afternoon. Rainfall is highest in June (214.3mm) and September (165.2mm).  Transmission of malaria in the area is intense and perennial with some seasonal variations. Most of the transmission (91.4%) occurs at night. Annual Biting Rates (ABRs) and Annual Entomological Inoculation Rates (AEIRs) in one study were reported to be 11,643 and 866, respectively (Abonuusum et al., 2011). The main malaria vectors are *Anopheles gambiae* and *A. funestus* and the main parasite species are *Plasmodium falciparum*, *P. malariae*, and *P. Ovale* (Abonuusum et al., 2011). |
| **Kilifi, Kenya** |
| Severe malaria cases were collected as part of on-going epidemiological studies of severe malaria at Kilifi District Hospital, Kenya. This hospital is situated in a rural area on the Kenyan Coast about 55km north of Mombasa. The local economy is predominantly rural, being based on subsistence farming of maize, cassava, cashew nuts, and coconuts as well as dairy cows and goats.  Malaria transmission in Kilifi is seasonal, generally coinciding with the long and short rains in October and May, respectively. Recent years have seen a significant decline in the rate of transmission from meso-endemic in the 1990’s to hypo-endemic transmission today. *P. falciparum* is the cause of the vast majority of malaria infections. The overall annual entomological inoculation rate (EIR) has been estimated at 1-100 (Mbogo et al., 1993; Mbogo et al., 2003). |
| **Muheza, Tanzania** |
| Severe malaria cases were recruited from the Teule Hospital in Muheza in the Tanga region of North East Tanzania. The dominant climate is warm and wet due to the influence of the Indian Ocean. However, during the hot season (December to March) the average temperature is approximately 30-32^O^C during the day and 26-29^O^C at night. During the cool season (May to October) temperatures are approximately 23-28^O^C in the day and 20-24^O^C at night. Another characteristic of the coastal climate is the high atmospheric humidity, which often goes up to 100% maximum and 65-70% minimum. Mean annual rainfall ranges from 600-800 mm. The coastal area is dominated by bush land, palm gardens, village cultivations and estates (mainly sisal). The upland plateaus are covered with bush land and shrub thickets interrupted by swampy low-lands and river swamps as well as village cultivations, estates and palm gardens.  The study villages lie at an altitude of 199-300 metres. Transmission of *Plasmodium falciparum* is intense (50-700 infected bites/person/year) and perennial, with two seasonal peaks (Maxwell et al., 2003). The community prevalence of *P. falciparum* in children aged 2-5 years in the study area was recorded as 88.2% in 2002 (Maxwell et al., 2003). |
| **Blantyre, Malawi** |
| The Malawi-Liverpool Wellcome Clinical Research Programme (MLW) together with the Blantyre Malaria Project (BMP) recruited severe malaria cases, healthy controls and parents from the Queen Elizabeth Central Hospital (QECH) in Blantyre, Malawi. Blantyre, a compact, densely populated city of 500,000 people, is situated at 1000m above sea level. Daytime temperatures average around 25^o^C, with considerable variation throughout the year. The climate is characterised by a cool, dry season (May-September) and a hotter rainy season (October - April). Malaria transmission is year-round, with considerable seasonal fluctuation and most new infections occur during the hot and wet season. Over 90% of human malaria infections are due to Plasmodium falciparum, while *5-10%* are P. malariae and 1-2% P. ovale. P. vivax is not present, owing to the almost universal presence of Duffy-negativity among the Malawian population (Skarbinski et al., 2012). The entomological inoculation rate (EIR) for P. falciparum in the city of Blantyre is estimated to be around one infective bite/person/year, but a high proportion of families make regular visits to nearby rural areas where the EIR is estimated to be greater than 100. |

**References for supplementary table 2**

Abonuusum A, Owusu-Daako K, Tannich E, May J, Garms R, Kruppa T. (2011) Malaria transmission in two rural communities in the forest zone of Ghana. *Parasitol Res* 108:1465-1471.

Maxwell CA, Chambo W, Mwaimu M, Magogo F, Carneiro IA, Curtis CF. (2003) Variation of malaria transmission and morbidity with altitude in Tanzania and with introduction of alphacypermethrin treated nets. *Malar J* 2:28.

Mbogo CM, Mwangangi JM, Nzovu J, Gu W, Yan G, Gunter JT, Swalm C, Keating J, Regens JL, Shililu JI, Githure JI, Beier JC. (2003) Spatial and temporal heterogeneity of Anopheles mosquitoes and Plasmodium falciparum transmission along the Kenyan coast. *Am J Trop Med Hyg* 68:734-742.

Mbogo CN, Snow RW, Kabiru EW, Ouma JH, Githure JI, Marsh K, Beier JC. (1993) Low-level Plasmodium falciparum transmission and the incidence of severe malaria infections on the Kenyan coast. *Am J Trop Med Hyg* 49:245-253.

Skarbinski J, Mwandama D, Wolkon A, Luka M, Jafali J, Smith A, Mzilahowa T, Gimnig J, Campbell C, Chiphwanya J, Ali D, Mathanga DP. (2012) Impact of indoor residual spraying with lambda-cyhalothrin on malaria parasitemia and anemia prevalence among children less than five years of age in an area of intense, year-round transmission in Malawi. *Am J Trop Med Hyg* 86:997-1004.
